# The effect of a stress and anxiety coping program on objective structured clinical examination performance among nursing students in shiraz, Iran

**DOI:** 10.1186/s12909-020-02228-9

**Published:** 2020-09-14

**Authors:** Sadaf Mojarrab, Leila Bazrafkan, Azita Jaberi

**Affiliations:** 1 Medical Education Department, Medical Education Development Centre, Shiraz University of Medical Sciences, Shiraz, Iran; 2 Clinical Education Research Centre, Education Developmental Centre, Shiraz University of Medical Sciences, Sina-Sadra Halls Complex, Neshat Ave, Shiraz, Postcode: 7134874689 Iran; 3 Community Based Psychiatric Care Research Center, School of Nursing and Midwifery, Shiraz University of Medical Sciences, Shiraz, Iran

**Keywords:** Anxiety, Nursing students, State-trait anxiety inventory

## Abstract

**Background:**

Evaluation of the competence and practical skills of nursing students, using the objective structured clinical examination (OSCE), is an integral part of the nursing education program. However, their performance could be negatively influenced by a significant level of stress and anxiety prior to the test. The present study aimed to evaluate the effect of an anxiety coping program on the OSCE performance level of first-year nursing students in Shiraz, Iran.

**Methods:**

The present quasi-experimental study was conducted among 76 nursing students; control group (*n* = 35) and intervention group (*n* = 41). To attain the study purpose, the intervention group received a pre-exam anxiety coping program that included relaxation and soothing techniques, diaphragmatic breathing training, and progressive muscle relaxation training accompanied by light instrumental music, while the control group received no intervention before the exam. Data collection instruments included a demographic characteristics form and State-Trait Anxiety Inventory (STAI) questionnaire. Demographic characteristics of the participants indicated an overall homogeneity within the study population. The STAI questionnaire was filled in before and after the OSCE and the results were compared with those of the control group. The data were analysed using SPSS software (version 22.0). *P* < 0.05 was considered statistically significant.

**Results:**

As a result of the anxiety coping program, a substantial reduction in the anxiety score (by 11.61 units) in the intervention group was observed. There was a significant difference in the pre- and post-exam anxiety scores between the control and intervention groups (*P* < 0.001). The anxiety coping program improved the examination results of nursing students in the final exam compared to the midterm results (an increase of 0.9487 units, *P* < 0.001).

**Conclusion:**

The anxiety coping program reduced the anxiety level among nursing students and improved their OSCE results. Our findings can be utilized to better evaluate clinical activities in different medical and paramedical groups. Moreover, educators can implement such coping programs prior to evaluations in order to effectively assess the knowledge, attitude, and performance of the students.

## Background

Effective evaluation of nursing students increases their motivation to elevate their clinical skills. It also permits nurse educators assess their teaching standards and to evaluate the effectiveness of the nursing curriculum [1]. The objective structured clinical exam (OSCE) is one of the most effective functional test methods to achieve these goals. The OSCE is typically multi-stationed, through which different types of skills and performance levels can be evaluated concurrently in a comprehensive and standardized manner. In addition, the OSCE allows the evaluation of non-cognitive attributes (perception, anxiety, confidence, preparedness), clinical competence, and psychosocial motor (motor and psychosocial skills) to track the students’ progress, which in turn not only increases students’ performance and confidence level but also enhances teaching enthusiasm among clinical educators [2]. Currently, the OSCE has been adopted as an official evaluation tool by most of the nursing and midwifery faculties [3–5].

It has been shown that the unfamiliarity of nursing students with the format and sequence of OSCE has been the main contributor to their increased level of anxiety and stress [6]. Various aspects of psychological stress, referred to as anxiety, among students prior to and during an examination have been studied [7]. Test anxiety is a biopsychosocial factor that affects both the academic performance of students and their well-being. In a moderate form, anxiety may positively contribute to the creativity and improved performance of students. However, intense anxiety may lead to inattention, shorter concentration spans, and reduced learning; leading to mistakes in classroom activities and a decrease in academic success [8]. Primary academic stressors for nursing students are exam preparation, taking the exam, and the anxiety of being evaluated [9]. While nursing students acknowledge the value of the clinical experience gained through an OSCE, however, they generally feel anxiety, loss of control, and pressure during the examination [10]. OSCE-related anxiety could be partly due to their unfamiliarity with the examination processes and requirements, which can be resolved using the familiarization (exposure) technique [5, 11, 12]. Successful implementation of the technique would ultimately improve student performance and their ability to succeed [6, 10].

The literature highlights the importance of stress reduction to allow students to maximize their learning and practical skills. Various techniques have been proposed to reduce the anxiety levels of nursing students such as music therapy, cognitive therapy, emotional freedom techniques (EFT), breathing techniques, mind cleansing, aromatherapy, and muscle relaxation [13, 14]. In this regard, a previous study in Turkey assessed the effect of music and EFT on the anxiety of nursing students during OSCE. However, no significant difference in the anxiety level between the control and intervention groups was observed [15]. Another study conducted in Ireland reported that the participation of nursing students in an OSCE familiarization workshop reduced their anxiety [16]. Simulated mock OSCE has been shown to reduce students’ anxiety [17]. Video presentations and pre-OSCE preparation have also been shown to improve students’ understanding of OSCE expectations, which in turn reduces the level of anxiety and stress [18]. Various studies in Iran have addressed OSCE-related anxiety among nursing students. A study assessed the effect of listening to the Holy Quran recitation on anxiety level and reported no significant difference in the level of anxiety between the control and intervention groups [19]. In a randomized controlled trial, another study showed the effectiveness of using standardized patients on the performance of Iranian nursing students in the physical examination of the abdomen [20]. However, to the best of our knowledge, there have been no studies in Iran that investigated the effect of interventions, such as anxiety coping program, and assessed their impact on anxiety reduction in nursing students before an OSCE.

Given the impact of the nature of anxiety in such tests and the need to deploy strategies that minimize anxiety and stress among nursing students, as well as the scarcity of such studies, the present study aimed to evaluate the effect of an anxiety coping program on the OSCE performance level of first-year nursing students in Shiraz, Iran.

## Methods

### Study design and participants

The present quasi-experimental study was conducted during October–December 2018 at Hazrat-e-Fatemeh Nursing and Midwifery Faculty, Shiraz University of Medical Sciences, Shiraz, Iran. The research goals were to quantify the levels of test anxiety associated with the OSCE among two groups of nursing students and to assess the effect of an anxiety coping program on the stress levels in the intervention group. The participants were recruited among nursing students enrolled in two consecutive academic calendar years; 2017–2018 and 2018–2019. Based on a simple random sampling method, 76 nursing students were recruited and divided into two groups, namely the control group and the intervention group. The control group (*n* = 35) included those enrolled during the academic calendar year 2017–2018. At the time of the present study, these students had completed the first-year and had already experienced the anxiety associated with the OSCE. The intervention group (*n* = 41) included freshly enrolled nursing students from the academic calendar year 2018–2019. These students had to take the OSCE as part of their curriculum. The anxiety coping program included a full explanation of the OSCE examination and its various stages, relaxation and soothing techniques, diaphragmatic breathing training, and progressive muscle relaxation training accompanied by light instrumental music.

### Measures

#### Socio-demographic information

Demographic characteristics of the participants included sex, age, marital status, kind of accommodation, diploma core curriculum, the city and year of the issued diploma, and the average score of the university entrance examination.

#### State-trait anxiety inventory (STAI)

The self-evaluation STAI questionnaire, developed by Spielberger in 1983, includes separate scales for measuring state (S-scale) and trait (T-scale) anxiety. In the present study, only the S-anxiety scale (STAI Form Y-1) was used. The questionnaire consisted of 20 items that evaluated how the participants felt at the time of responding to each item. Note that 10 items were associated with the anxiety-present (items 3, 4, 6, 7, 9, 12, 13, 14, 17, 18) and the remaining items were associated with the anxiety-absent (items 1, 2, 5, 8, 10, 11, 15, 16, 19, 20). The intensity of the participants’ feelings was rated on a 4-point Likert scale: (I) not at all, (II) somewhat, (III) moderately so, and (IV) very much so. The anxiety-present items were scaled from 1 to 4 such that higher scores indicated the presence of a high level of anxiety. However, the anxiety-absent items were scaled in reverse from 4 to 1. The total score for the STAI Form Y-1 ranged from a minimum of 20 to a maximum of 80.

The reliability and validity of the questionnaire ranged between 0.86–0.95 and 0.65–0.75, respectively [21]. The Persian version of the questionnaire was developed by Mahram and its reliability for the anxiety-present and anxiety-absent (Cronbach’s alpha 0.908 and 0.902, respectively) was confirmed. Additionally, the reliability of the questionnaire was confirmed by calculating the proportion of the true variance over the obtained variance (0.945). Furthermore, the validity of the questionnaire was confirmed using the concurrent validity method. Two other studies also reported 90 and 93% reliability of the questionnaire, respectively [22].

#### Procedure

Upon obtaining formal approval from the university authorities, a list of the nursing students for the 2017–2018 (control group) and 2018–2019 (intervention group) academic calendar years was obtained. The data associated with the control group were retrieved from the university database for comparison with the intervention group. Before the study, the research goals and procedures were explained after which written informed consent was obtained from the participants in the intervention group. At the beginning of the academic term, the participants in the intervention group were requested to fill in the demographic information form and the STAI Form Y-1 questionnaire. Subsequently, they followed an anxiety coping program that included relaxation and soothing techniques, diaphragmatic breathing training, and progressive muscle relaxation training accompanied by light instrumental music.

The initial research design included a total of six intervention sessions prior to the OSCE; three sessions per week, during 2 weeks, each of 40 min duration. However, due to the tight syllabus schedule of the university program and objection by the education deputy of the university, eventually only one intervention session was conducted prior to the OSCE. Nonetheless, the quality of the session was high and fully informed the students about the purpose of the OSCE examination and its various stages. The explanation was given by the first author with the help of a slideshow and video presentation showing former students taking the OSCE. In addition, to reduce anxiety, various relaxation techniques were described by an experienced academic psychiatric nurse. Prior to taking the OSCE, the participants of the intervention group gathered in a quiet and calm room and were requested to exercise the relaxation techniques while light instrumental music was being played. After completing the OSCE, they filled in the STAI Form Y-1 questionnaire for the second time.

### Statistical analysis

The data were analysed with the SPSS software (version 22.0) using descriptive and inferential statistics. Descriptive data were expressed as mean, standard deviation, number, and percentage. To compare the homogeneity of variables between the two groups, the Chi-square test and independent t-test were used. To compare the anxiety levels in both groups pre- and post-intervention, the paired sample t-test and independent t-test were used. *P* < 0.05 was considered statistically significant.

### Ethical considerations

The present study was approved by the Ethics Committee of Shiraz University of Medical Sciences, Shiraz, Iran (code: IR.SUMS.REC.1389.014). Approval from the Dean of Hazrat-e-Fatemeh Nursing and Midwifery Faculty was obtained to access the relevant data of the enrolled nursing students. The participants were informed about the research goals and procedures. Also, the confidentiality of any disclosed information was guaranteed and voluntary participation was emphasized. Written informed consent was obtained from the participants.

## Results

Demographic characteristics of the participants (Table 1) indicated an overall homogeneity within the study population.

**Table 1:** Socio-demographic characteristics of participants

Variables		Control group		Intervention group		*P* value
		N	%	N	%	
Sex	Female	19	54.3	22	53.7	0.956
	Male	16	45.7	19	46.3	
Marital status	Single	30	85.7	39	95.1	0.157
	Married	5	14.3	2	4.9	
Diploma type	Natural sciences	31	88.6	35	85.4	0.803
	Nursing assistant	2	5.7	4	9.8	
	Others	2	5.7	2	4.9	
Place of issue of the diploma	Shiraz	8	22.9	12	29.3	0.165
	Fars province	21	60.0	16	39.0	
	Others	6	17.1	13	31.7	
University entry grade	<3000	15	42.9	20	48.8	0.201
	3000-5000	8	22.9	14	34.1	
	>5000	12	34.3	7	17.1	
Accommodation	University dormitory	27	77.1	28	68.3	0.390
	Private	8	22.9	13	31.7	

*Chi-square test

A good proportion of the participants obtained excellent ranking in the nationwide university entry examination. This highly competitive examination is ranked based on the total number of students wishing to enter university; those with a lower ranking have a higher chance of being accepted. The results showed that 42.9 and 48.8% of the participants in the control and intervention group, respectively, obtained a ranking in the category below 3000. Such excellent performance is not only indicative of their academic skills, but it also illustrated their resilience to anxiety. There was no significant difference between the groups in terms of the university entry ranks (*P* > 0.05).

The results of the SATI score and the effect of the anxiety coping program are shown in Table 2. The mean age of the participants in the control and intervention group was 21.9 ± 3.55 and 19.98 ± 2.04, respectively. There was no significant difference in the mean age between the groups (*P* > 0.05).

**Table 2:** The effect of the intervention program on anxiety scores and examination results

Variables		Control group	Intervention group	*P* value*
		Mean±SD		
Age		21.9±3.55	19.98±2.04	0.710
Anxiety score	Pre-exam	42.03±9.618	47.78±10.492	0.016
	Post-exam	50.349±9.459	36.17±12.595	0.001
	*P* value**	0.001	0.001	
Exam result	Midterm	8.0694±0.68179	7.2093±1.05615	0.001
	Final	7.7614±0.89470	8.1580±1.07190	0.087
	*P* value**	0.001	0.001	

*Independent t-test, **Paired t-test

The pre- and post-exam anxiety scores showed the positive effect of the intervention. The results showed an increased anxiety score (8.31 unit) in the control group after the OSCE. The results of the paired t-test showed a significant difference (*P* < 0.001) in the pre- and post-exam scores of the control group. However, in the intervention group, a substantial reduction in the anxiety score (11.61 unit) was observed. The results of the paired t-test showed a significant difference in the pre- and post-exam scores between the groups (*P* < 0.001). A comparison between the control and intervention group, using the independent t-test, showed a significant difference in the anxiety score between the groups in the pre-exam (an increase of 5.75 units,(*P* < 0.05) and post-exam (a decrease of 14.12 units, *P* < 0.001) stages. These results confirmed the effectiveness of the anxiety coping program.

Based on the paired t-test, the final examination results obtained by the participants in the control group showed a slight decline (a decrease of 0.308 units, *P* < 0.001) compared to the midterm results. However, in the intervention group, the positive effect of the anxiety coping program resulted in an improvement in the final exam results compared to the midterm results (an increase of 0.9487 units, P < 0.001). The results of the independent t-test between the groups showed improved final examination results (an increase of 0.396, *P* > 0.05) in the intervention group.

## Discussion

The present study aimed to determine the effect of an anxiety coping program on nursing students’ performance in OSCE in Shiraz, Iran. The anxiety score was measured pre- and post-examination in both the control and intervention groups.

The results showed an increased anxiety score among the nursing students in the control group after the OSCE. We found that the anxiety coping program had a positive effect on the average anxiety score of nursing students during the OSCE. A similar study conducted by Yalcin in Turkey also reported a higher anxiety score among medical students after taking part in an OSCE [23]. Although nursing and medical students are different target populations, studying different subjects, however the anxiety caused by the OSCE is similar in both groups. Yalcin showed that the anxiety level in the intervention group was reduced while it had increased in the control group, indicating the importance of an anxiety coping program in medical students before the OSCE. In line with the study by Yalcin, we also found a significant positive effect of the anxiety coping program in the intervention group pre- and post-examination. Moreover, it was shown that such coping programs could reduce anxiety among students immediately after the intervention, on the day of an OSCE, and 3 months after the test. In another study, Dunne and colleagues stated that anxiety coping programs before an OSCE could effectively control the anxiety level among nursing students [16]. Other researchers have also indicated the positive effect of psychological intervention in reducing anxiety levels, demotivation, and mental stress among students [24]. While OSCE is an excellent opportunity for students to enhance their clinical skills, it involves many challenges and issues such as anxiety [25]. Therefore, interventions to prepare students to study and readiness for an OSCE could reduce the level of anxiety before the examination [26]. Such coping programs could help students to manage their anxiety and experience less stress. Other intervention methods such as meditation have been successfully used to reduce anxiety. A study in Thailand among nursing students at various universities reported the effectiveness of meditation in reducing the anxiety level during clinical examinations [27].

The results of the present study showed that our anxiety coping program led to an improvement of the average examination results of the nursing students. In line with a study by Yusefzadeh, we found that the intervention group obtained significantly better exam results compared to the control group. It has been shown that the anxiety associated with an OSCE could negatively affect the thinking process and subsequently negatively affect the performance of the students. Also, essential parameters that enhance performance (knowledge, attitude, and psychomotor) could be impaired by anxiety [28].

A study conducted on anxiety among cosmetic surgery students during their OSCE reported that it negatively affected their performance [29]. In line with these findings, the results of the present study showed that the intervention group obtained better examination results compared to the control group. Another study investigated factors that caused anxiety during an OSCE [30]. Among different factors, it was reported that students attending lectures with a negative attitude were more prone to anxiety during the OSCE. In addition, familiarization with the OSCE process and simulated mock OSCE were also suggested as effective methods to increase students’ self-confidence and consequently reduce anxiety during the actual test [31].

Considering the importance of critical thinking skills in nursing, it is inevitable to use OSCE since it encourages critical thinking in nursing students [32]. Since OSCE is a formal practical examination, it is natural to anticipate high levels of anxiety among students taking the test. As reported by other studies, an OSCE caused more anxiety compared to any other assessment format. Such high levels of anxiety could be due to the fact that the students are individually and visibly under scrutiny, and the test at each OSCE station is done only once, is irreversible, and there is no opportunity for corrective actions. In the present study, before the actual OSCE, efforts were made to familiarize the participants with the OSCE process through a slideshow and video presentations.

### Application of research findings

The main objective of the present study was to reduce the anxiety of nursing students before taking the OSCE in order to empower them to better recognize anxiety and utilize their skills in managing it. The anxiety coping program included relaxation and soothing techniques, diaphragmatic breathing training, and progressive muscle relaxation training accompanied by light instrumental music. Since an OSCE is used in various medical disciplines to assess the clinical skill performance and competence of students, the findings of the present study are applicable in the fields of clinical practice, management, and education.

- Clinical practice: The positive effect of a coping program to reduce anxiety and improve exam results has been demonstrated. Our findings can be utilized to better evaluate clinical activities in different medical and paramedical groups.
- Management: The findings can be used by policymakers and educational planners to make effectvie use of the OSCE by including coping programs to help students to overcome anxiety-related issues.
- Education: Considering the positive effect of the coping program and the improved examination results, educators can implement such coping programs prior to evaluations in order to effectively assess the knowledge, attitude, and performance of the students.

### Recommendations for future studies

To complement the findings of the present study, it is recommended to conduct the following assessment studies.

- The effect of anxiety coping program on the anxiety level in paramedical students and on their OSCE examination results.
- The effect of anxiety coping program on the educational motivation, learning satisfaction, and academic performance of nursing students after an OSCE.
- The effect of mock OSCE tests on anxiety and examination results of nursing students.
- Identification of anxiety predictive factors in nursing students.

### Study limitations

The main limitation of the present study was the participation of students from a single university and medical discipline (nursing) which limits the generalizability of our findings. Furthermore, the anxiety coping program was performed during a limited period and there was no follow-up on the state of anxiety among students. It is recommended that future studies include different medical disciplines across different universities and to extend the duration of the intervention as well as the number of follow-up visits.

## Conclusion

The findings of the present study indicated that an anxiety coping program reduced the anxiety level among nursing students and improved their OSCE results. Educational planners should consider offering a curriculum to reduce anxiety and help students to improve their examination results. It is recommended to conduct future studies over a longer period and with follow-ups of the students’ academic performance.

## Data Availability

The datasets generated and analysed during the current study are not publicly available due to confidentiality of the identity of the participants. Data are however available from the authors upon reasonable request.

## Abbreviations

OSCE: Objective Structured Clinical Examination
STAI: State-Trait Anxiety Inventory

## References

[1] Billings DM, Faan ER, Halstead JA (2019). Teaching in nursing e-book: a guide for faculty: Elsevier health sciences.

[2] Caballero C, Creed F, Gochmanski C, Lovegrove J (2012). Nursing OSCEs: a complete guide to exam success: OUP Oxford.

[3] Obizoba C (2018). Mitigating the challenges of objective structured clinical examination (OSCE) in nursing education: a phenomenological research study. Nurse Educ Today.

[4] Selim AA, Ramadan FH, El-Gueneidy MM, Gaafer MM (2012). Using objective structured clinical examination (OSCE) in undergraduate psychiatric nursing education: is it reliable and valid?. Nurse Educ Today.

[5] Bani-issa W, Al Tamimi M, Fakhry R, Al TH (2019). Experiences of nursing students and examiners with the objective structured clinical examination method in physical assessment education: a mixed methods study. Nurse Educ Pract.

[6] Johnston AN, Weeks B, Shuker M-A, Coyne E, Niall H, Mitchell M (2017). Nursing students' perceptions of the objective structured clinical examination: an integrative review. Clin Simul Nurs.

[7] Sulistiowati NMD, Septiadi WN, Nopriani LP (2017). Anxiety level analysis of nursing student who took objective structured clinical examination (Osce) using face temperature distribution based on thermal imaging. Indones Nurs J Educ Clin (INJEC).

[8] Torabizadeh C, Bostani S, Yektatalab S (2016). Comparison between the effects of muscle relaxation and support groups on the anxiety of nursing students: a randomized controlled trial. Complement Ther Clin Pract.

[9] Yıldırım N, Karaca A, Ankaralı H, Açıkgöz F, Akkuş D (2016). Stress experienced by Turkish nursing students and related factors.

[10] Stunden A, Halcomb E, Jefferies D (2015). Tools to reduce first year nursing students' anxiety levels prior to undergoing objective structured clinical assessment (OSCA) and how this impacts on the student's experience of their first clinical placement. Nurse Educ Today.

[11] Muldoon K, Biesty L, Smith V (2014). ‘I found the OSCE very stressful’: student midwives' attitudes towards an objective structured clinical examination (OSCE). Nurse Educ Today.

[12] Fidment S (2012). The objective structured clinical exam (OSCE): a qualitative study exploring the healthcare student’s experience. Stud Engagement Exp J.

[13] Patterson SL (2016). The effect of emotional freedom technique on stress and anxiety in nursing students: a pilot study. Nurse Educ Today.

[14] Ince S, Çevik K (2017). The effect of music listening on the anxiety of nursing students during their first blood draw experience. Nurse Educ Today.

[15] İnangil D, Vural PI, Doğan S, Körpe G (2020). Effectiveness of music therapy and emotional freedom technique on test anxiety in Turkish nursing students: a randomised controlled trial. Eur J Integr Med.

[16] Dunne K, Moffett J, Loughran ST, Duggan V, Campion DP (2018). Evaluation of a coaching workshop for the management of veterinary nursing students’ OSCE-associated test anxiety. Ir Vet J.

[17] Young I, Montgomery K, Kearns P, Hayward S, Mellanby E (2014). The benefits of a peer-assisted mock OSCE. Clin Teach.

[18] Massey D, Byrne J, Higgins N, Weeks B, Shuker M-A, Coyne E (2017). Enhancing OSCE preparedness with video exemplars in undergraduate nursing students. A mixed method study. Nurse Educ Today.

[19] Jafaripour H, Momeni H, Salehi A, Tavan B, Mahmoodi A (2019). The effect of Holy Quran on anxiety caused by the OSCE in Khomein University of medical sciences students. Complement Med J Arak Univ Med Sci.

[20] Jaberi A, Momennasab M (2019). Effectiveness of standardized patient in abdominal physical examination education: a randomized, Controlled Trial. Clin Med Res.

[21] Spielberger CD (1983). STAI state-trait anxiety inventory for adults form Y: review set; manual, test, scoring key: mind garden.

[22] Beiranvand S, Hosseinabadi R, Fatemeh S (2017). An assessment of nursing and midwifery student Veiwwpoin, performance, and feedback with an objective structured clinical examination (OSCE). J Nurs Educ.

[23] Yalcin BM, Unal M, Pirdal H, Karahan TF. The effect of a stress and anxiety coping programme on objective structured clinical exam performance in medical students, a randomised clinical trial. Educ Psychol. 2015;10:1–14.

[24] Saravanan C, Kingston R (2014). A randomized control study of psychological intervention to reduce anxiety, amotivation and psychological distress among medical students. J Res Med Sci.

[25] Harden RM (2016). Revisiting ‘assessment of clinical competence using an objective structured clinical examination (OSCE)’. Med Educ.

[26] Yusefzadeh H, Iranagh JA, Nabilou B. The effect of study preparation on test anxiety and performance: a quasi-experimental study. Adv Med Educ Pract. 2019;10:245–51. 10.2147/AMEP.S192053 PMC6524999 31191073

[27] Ratanasiripong P, Park JF, Ratanasiripong N, Kathalae D (2015). Stress and anxiety Management in Nursing Students: biofeedback and mindfulness meditation. J Nurs Educ.

[28] Khan KZ, Ramachandran S, Gaunt K, Pushkar P (2013). The objective structured clinical examination (OSCE): AMEE guide no. 81. Part I: an historical and theoretical perspective. Medical teacher.

[29] Zhang N, Rabatsky A (2015). Effects of test stress during an objective structured clinical examination. J Chiropr Educ.

[30] Kim KJ (2016). Factors associated with medical student test anxiety in objective structured clinical examinations: a preliminary study. Int J Med Educ.

[31] Emery AW, Rose-Innes E (2018). Benefits of a peer-led mock-OSCE.

[32] Shirazi F, Heidari S (2019). The relationship between critical thinking skills and learning styles and academic achievement of nursing students. J Nurs Res.

